# Pharmacist-led medication-related needs assessment in rural Ghana

**DOI:** 10.1186/2193-1801-2-163

**Published:** 2013-04-16

**Authors:** Kyle John Wilby, Jill Lacey

**Affiliations:** 1 Assistant Professor - Clinical Pharmacy and Practice, College of Pharmacy, Qatar University, PO Box 2713, Doha, Qatar; 2 Clinical Pharmacist, Saskatoon Cancer Centre, 20 Campus Drive, Saskatoon, Saskatchewan S7N 4H4 Canada

**Keywords:** Pharmacy, Public health, Needs assessment, Medication

## Abstract

Access to both essential and non-essential medications is increasing worldwide. While increased drug access is a positive development, many countries lack the infrastructure for appropriate distribution, administration, and monitoring of drug therapy. The objective of this study was to assess medication and pharmacy-related needs in the rural Ashanti Region of Ghana and to determine barriers of achieving optimal health outcomes in this region. Qualitative domains and associated themes were identified by observations from integration into community culture and from conduction of semi-structured interviews with local community leaders, health workers, or those with knowledge of health-related issues. Eight semi-structured interviews were completed and four thematic domains were identified; access to care, resource shortages, medication safety, and education/training. Barriers and challenges identified under each thematic domain included (but were not limited to) availability of clean water sources, shortages of medications and diagnostic equipment, financial considerations, misunderstanding of medication indications and directions for use, and shortages of qualified pharmacy or dispensary staff. Most respondents also expressed a need for continuing education and training of healthcare personnel. It can be concluded that there is a need for development of health services related to medications. Locally supported interventions and future research should focus on barriers and challenges identified from the thematic domains.

## Background

Access to both essential and non-essential medications is increasing worldwide, largely facilitated by global health partnerships and international policy (Ngoasong p 2009). Expansion of medication use creates both opportunities and challenges for health care systems. While increased drug access is a positive development, many countries lack the infrastructure for appropriate distribution, administration, and monitoring of drug therapy (Bhargava 2005). Additionally, shortages of trained pharmacists and pharmacy technicians may further increase demands placed on already stressed systems (Smith 2004). In order to develop solutions to these challenges, region-specific medication-related needs assessments are required to characterize strengths and weaknesses of current operations.

Ghana is a country located in Western Africa with an estimated population of 25 million people (World Bank 2012). Tertiary health services are available in urban centres, with rural centres mostly relying on small publically funded clinics. In the rural areas, patients usually obtain medications from public clinics, or independently owned and operated chemical shops. Medications are dispensed by many types of healthcare professionals, including pharmacists, pharmacy technicians, nurses, and nursing students. However, it is common for professional staff to be absent in pharmacies and even more so in chemical shops (Smith 2004). Situation-based needs assessments may be useful, especially in these rural areas, to help governments, non-governmental organizations, and international volunteer organizations target specific strategies relating to the medication needs of these regions.

The objective of this study was to assess medication and pharmacy-related needs in the rural Ashanti Region of Ghana and to determine barriers to achieving optimal health outcomes in this region.

## Results

### Description of study sites

One town had a population of approximately 5000 people. It contains two medical clinics, one public and one that is privately funded. The public clinic serves the town population and that of the surrounding villages. An estimated total population of all people within the region is 10 000 people. The clinic provides basic medical services, including maternal-fetal medicine, vaccinations, nutrition counseling, and treatment of infectious diseases. Complex cases or patients requiring diagnostic imaging or surgery are referred to larger health centers in the district. Medications regularly available at the clinic include analgesics, oral antibiotics, intravenous rehydration solutions, vitamins, and antimalarials. They are dispensed by a pharmacy technician. Clinic staff provides outreach vaccination and first aid services to surrounding villages but villagers are expected to travel to town if other medical services are needed. The private clinic provides similar services. Outpatient medications can be purchased without a prescription from four chemical shops located within town. These shops sell non-narcotic analgesics, antibiotics, antimalarials, and herbal products. Chemical shop staff rarely has pharmacist or pharmacy technician training.

The other location was a small village located five kilometers from the major town. Its population is approximately 300 people. There are no health services offered in the village but the outreach team from the clinic in town travels to this village usually once per month for outreach visits. There is no direct transport between the two, except once per week when cars transport people to and from the market in the larger town.

### Semi-structured interviews

Eight community members were identified to complete semi-structured interviews and informed consent was obtained. Individual professions/community roles were as follows: 1 laboratory technician/diagnostician, 1 international volunteer coordinator, 3 chemical shop owners, 1 nursing assistant, 1 pharmacy technician, and 1 teacher. All interviewees lived and worked within the two centres described.

### Assessment of community needs

Four common domains encompassing medication-related challenges were identified. These domains were ‘access to care’, ‘resource shortages’, ‘medication safety’, and ‘education/training’. ‘Access to care’ encompassed multiple identified challenges, including healthcare worker shortages, geographical and transportation logistical challenges, and unaffordable costs of care. ‘Resource shortages’ comprised of challenges associated with limited access to clean water, electricity, medications, diagnostic testing, and equipment necessary for monitoring of chronic diseases. ‘Medication safety’ was commonly discussed as a broad category that encompassed knowledge and understanding of medications, adverse effects, and cultural beliefs with respect to drug use. Most healthcare workers discussed ‘Education/training’ as a barrier to providing high quality pharmacy services, in addition to shortage of healthcare personnel.

## Discussion

This study assessed medication-related needs of two rural centres in the Ashanti Region of Ghana through observations and the use of semi-structured interviews. Four domains were identified that allow for further analysis of the factors affecting the determinants of health in these regions. These domains are similar to challenges previously speculated for the country as a whole (Smith 2004).

‘Access to care’ and ‘resource shortages’ were the most commonly identified domains from all interviews. While it was initially speculated that access to medications or healthcare personnel might be barriers, it was found that access to water, electricity, and sanitation precluded these concerns. For example, clinic staff expressed concerns regarding the stability of medications and vaccines that require refrigeration. Frequent electrical outages put supplies at risk of spoilage, in an already rationed environment. Additionally, a lack of potable water and adequate sanitation facilities put community members at risk of exposure to disease.

With respect to pharmacy services and medications, clinical pharmacy services were reported to be rare with most patients receiving basic counseling instructions from dispensing staff with a variety of training. The closest practicing pharmacist was located 10 kilometers away from the larger town. Medications available in town included antimalarials, oral antibiotics, rehydration solutions, cough and cold products, analgesics, and a variety of herbal products and supplements. Antihypertensives and oral hypoglycemic agents were available but in short supply. The development of clinical pharmacy services through education and training and optimizing the use of medications would benefit these communities.

The cost of medications and healthcare visits was mentioned as a barrier to health in a number of interviews. While patients are required to pay for services and medications, a national health insurance scheme has been recently developed that allows for coverage/subsidies of clinic visits and medications. As described by the respondents, uptake of this scheme has been low. Barriers identified through the interviews included misunderstanding of program objectives, mistrust of previous initiatives, and inexperience with insurance policies. Education regarding the national health insurance scheme is urgently needed, in order to ensure continued success of the program.

‘Medication safety’ was identified as a need for improvement of health services in these communities. It was specifically mentioned that development of medication adverse effects results in many discontinuations of treatment, due to misunderstanding that some adverse effects are to be expected. Also, dosage instructions provided are quite minimal and not always in the patient’s primary language. This may lead to incorrect administration of medications. Advancement of clinical pharmacy services may circumvent these issues by addressing patient concerns through 1-on-1 counseling sessions and education initiatives.

Finally, ‘education and training’ was the last domain identified. Participants commented on lack of training and continuing education opportunities for healthcare workers, including those responsible for providing medications. It has been identified before that personnel with a variety of training work in healthcare settings and that this creates challenges for provision of quality care (Smith 2004). Specific to pharmacy, an emphasis on the development of clinical services (counseling, identification of drug therapy problems, and monitoring patients for efficacy and toxicity) and continuing education with respect to drug information may be helpful to address this need. Internal and external programs should focus on continuing education of dispensary staff (pharmacists, pharmacy technicians, chemical shop workers, nurses, etc.) to enhance the optimal use of medications.

## Conclusions

This study identified four common domains, as part of a medication-related needs assessment conducted in rural Ghana. While the points discussed can be used to direct future interventions and research, several limitations should be noted. This study was limited to these two villages in Ghana and results should not be generalized to all rural populations without adequate assessment. Also, a small convenience sample was used for this analysis and the responses obtained may not be reflective of the entire regional population. However, as all domains were identified in the majority of responses, it is unlikely this would cause significant bias.

In conclusion, medication-related needs assessments will continue to be important for governments and international health organizations as access to medications improves worldwide. Locally supported interventions and future research are needed to address challenges associated with the identified domains of access to care, resource shortages, medication safety, and education/training.

## Methods

Ethics approval for this study was obtained from the ethics review board at the University of Saskatchewan, Saskatoon, Canada. A formal ethical review process was not available for the particular region in Ghana at the time of the study but all documents, consent forms, and study procedures were approved by the director or owner of each workplace as well as the chief of each village. Data was gathered through two different methods; observation of community practices and semi-structured interviews with community members and healthcare professionals. Semi-structured interviews utilized a series of leading questions (Table 1) to prompt the participant to discuss topics and issues of relevance. Each interview lasted approximately 20–30 minutes. The investigators integrated into the targeted communities by contributing towards community development efforts within the education and healthcare systems. Observations about healthcare and medication utilization were documented and used to direct the semi-structured interviews.

**Table 1:** **Leading questions for semi-structured interviews**

	Question
1	What are the major health issues and challenges affecting Nsuta and/or Jansa?
2	How do people commonly obtain health services? How do they obtain medications and/or pharmacy services?
3	Are there any health services you think would be beneficial? What would they be? Who would obtain them?
4	What kind of help would be useful for improvement of services? Do you think any help is needed from outside of Ghana?

Participants for the semi-structured interviews were a convenience sample of community members with expertise and/or knowledge of healthcare related issues. Subjects were included if they were living or working within the regions of interest. Subjects were excluded if they were not part of the communities being assessed. Informed consent was obtained prior to initiating each interview. If subjects could not read English, the consent form was translated verbally into the subject’s understood dialect.

All interviews were conducted in person using predefined questions as a guide (Table 1). Participants were also encouraged to discuss their experiences with respect to healthcare as desired. The interviews were conducted in English but translators were available if necessary. Each interview was recorded using a handheld recording device. Upon completion of the interview, one investigator created a written transcript. Each subject was shown or read the transcript and was allowed to add, modify, or delete contents. The final transcripts required approval by the subject prior to inclusion in the study.

Two investigators independently assessed each transcript and identified commonalities from the content discussed. Results were compared and discrepancies were solved through discussion and agreement.

## Authors’ information

KW completed his education in Canada, has extensive previous experience working in Ghana and other parts of sub-Saharan Africa, and is now in an academic role in the Middle East.
